# Enhanced skin cancer classification using modified efficientNetV2L with adaptive early stopping mechanism

**DOI:** 10.1038/s41598-025-22228-3

**Published:** 2025-11-03

**Authors:** Chandrasekar Venkatachalam, Shanmugavalli Venkatachalam, Arunkumar Balakrishnan

**Affiliations:** 1 Department of Computer Science and Engineering, Faculty of Engineering, Jain (Deemed to be university), Bangalore, 562112 Karnataka India; 2 Manipal Institute of Technology Bengaluru, Manipal Academy of Higher Education, Manipal, India; 3 Manipal Institute of Technology Bengaluru, Manipal Academy of Higher Education, Manipal, India

**Keywords:** Skin cancer classification, EfficientNetV2L, Deep learning, Earlystopping, Callback mechanism, Computational biology and bioinformatics, Engineering, Mathematics and computing

## Abstract

The accurate classification of skin cancer types is a critical task in medical diagnostics, requiring robust and reliable models to distinguish between various skin lesions. Despite advancements in deep learning, developing models that generalize well to unseen data remains a challenge. Current methodologies primarily utilize convolutional neural networks (CNNs) for image classification tasks, leveraging architectures such as ResNet, VGG, and Inception. These models have shown promise in improving classification accuracy for skin cancer detection. However, existing models often face limitations, including overfitting to the training data and difficulty in handling imbalanced datasets. This results in decreased performance on validation and test datasets, reducing their practical applicability in clinical settings. Additionally, these models may lack the fine-grained discrimination required to accurately classify a diverse range of skin lesion types. To address the limitations of traditional CNN-based approaches, we propose a novel model based on the EfficientNetV2L architecture, optimized for skin lesion classification. Our approach introduces adaptive early stopping and learning rate callbacks to enhance generalization and prevent overfitting. Trained on the ISIC dataset, the model achieved a high classification accuracy of 99.22%, demonstrating robustness across various lesion types. This work contributes a powerful, efficient, and clinically relevant solution to the field of automated skin cancer diagnosis.

## Introduction

 Skin cancer is the most common cancer in the United States, with over 5 million new cases diagnosed each year. It develops when DNA in skin cells becomes damaged, causing them to grow uncontrollably. There are various forms of skin cancer, each differing in characteristics and associated risks^1^. This article provides an overview of skin cancer and delves into some of the more common types, such as actinic keratosis, vascular lesions, melanoma, and pigmented benign keratosis^2^.

### Actinic keratosis

Actinic keratosis (AK) is a common skin condition caused by prolonged exposure to ultraviolet (UV) radiation from the sun or tanning beds. It typically appears as rough, scaly patches on sun-exposed areas like the face, ears, and hands. These lesions can vary in color from pink to brown and may feel dry or crusty^3^. While actinic keratosis itself is not cancerous, it can potentially progress to squamous cell carcinoma if left untreated. Early detection and treatment, which may include topical medications, cryotherapy, or laser therapy, are important to prevent progression and maintain skin health^4^.

### Vascular lesions

Vascular lesions are abnormal clusters of blood vessels near the skin’s surface. They can present as red, purple, or pink discolorations on the skin and are often referred to as birthmarks or vascular malformations. While most vascular lesions are benign, some types, such as hemangiomas, can be associated with an increased risk of skin cancer.

### Melanoma

Melanoma is a serious form of skin cancer that begins in the melanocytes, the cells responsible for producing skin pigment. It is less common than other skin cancers but can be more aggressive and prone to spreading if not caught early. Melanoma often arises from a new or changing mole and is linked to excessive UV radiation exposure. Early detection through regular skin checks is crucial, as treatments vary from surgery to immunotherapy and targeted therapies, depending on the cancer’s stage and spread^5^.

### Pigmented benign keratosis

Pigmented benign keratosis, also known as seborrheic keratosis, is a common non-cancerous skin growth characterized by its pigmented, wart-like appearance. These lesions typically develop as people age and are usually harmless, presenting as flat or raised spots with a range of colors from light brown to black^6^. While they are not a sign of skin cancer, they can occasionally be mistaken for malignant conditions^7^. Regular monitoring and, if necessary, removal or biopsy can help ensure accurate diagnosis and maintain skin health.

### Squamous cell carcinoma

Squamous cell carcinoma, a type of skin cancer prevalent on sun-exposed areas like the face, ears, neck, lips, and hands, requires early detection for effective treatment. Symptoms include persistent, scaly patches with rough surfaces, firm red nodules, non-healing sores, wart-like growths that may bleed, and changes in mole appearance^8^.

### Basal cell carcinoma (BCC)

Basal Cell Carcinoma (BCC) is the most common type of skin cancer, originating in the basal cells of the skin’s outer layer^9^. It typically appears as a small, pearly bump or a scaly, reddish patch and is commonly found on sun-exposed areas such as the face and neck. BCC usually grows slowly and rarely spreads to other parts of the body, but if left untreated, it can cause local tissue damage. Early detection and treatment, often through surgical removal or topical therapies, are essential for effective management and to prevent recurrence^10^.

### Seborrheic keratosis (SK)

Seborrheic Keratosis (SK) is a benign skin growth commonly found in older adults, requiring accurate diagnosis to distinguish from other skin conditions, including cancer. Key characteristics include raised or flat growths varying in color (from white to dark brown or black) with a “stuck-on” or warty appearance. SKs are not caused by sun exposure and are more prevalent with age. Treatment is often unnecessary but may involve removal for cosmetic reasons or if the growth becomes irritated or itchy, using methods such as cryotherapy, curettage, or laser therapy.

### Dermatofibroma

Dermatofibroma, also known as fibrous histiocytoma, is a benign skin growth that commonly appears as a hard, raised nodule on the skin. While dermatofibromas are usually harmless, they can occasionally mimic skin cancer due to their appearance.

### Nevus

**Fig. 1:**
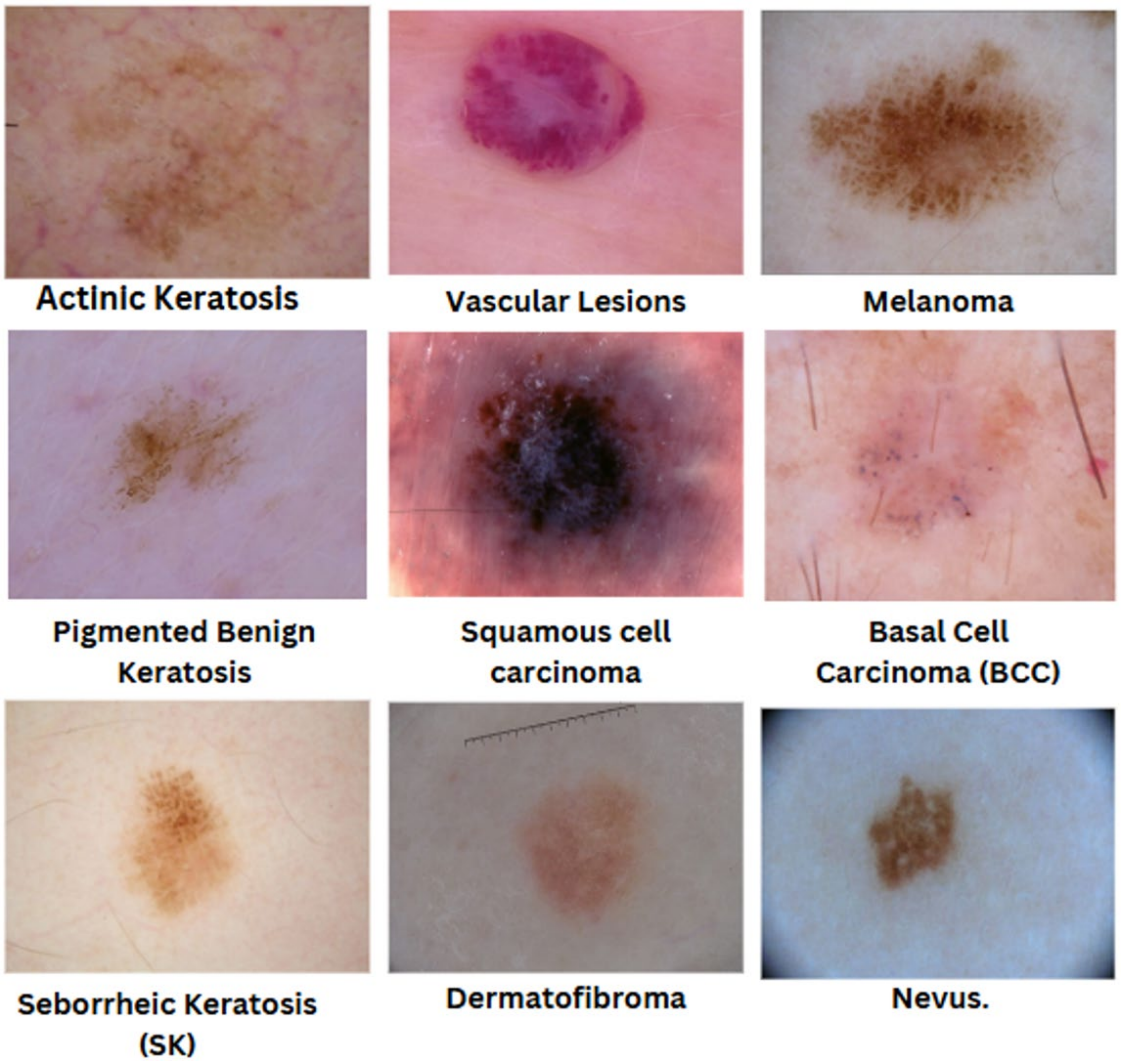
Sample of all type of skin cancer.

A nevus, commonly known as a mole, is a benign skin growth that consists of clusters of melanocytes, the cells responsible for skin pigmentation. Nevi can appear as flat or raised spots and vary in color from pink to brown or black. They are usually harmless and often present from birth or develop during childhood. While most nevi are benign, changes in their appearance, such as size, shape, or color, can sometimes indicate a risk of skin cancer. Regular skin checks are important for monitoring any changes and ensuring skin health. Figure 1 depicts all kinds of skin cancer.

Recent advances in deep learning have substantially improved medical image classification. For example, Khan et al.^35^ proposed a grid search–optimized multi-CNN ensemble for automated cervical cancer diagnosis, while Rehman et al.^36^ developed FOLC-Net, a federated lightweight model for MRI-based disease detection. Similarly, Asif et al.^37^ introduced ShallowMRI, a lightweight CNN with attention mechanisms for brain tumor classification. These works collectively demonstrate the effectiveness of optimized architecture, ensemble methods, and lightweight models in enhancing diagnostic performance across diverse medical imaging domains.

While convolutional neural networks like ResNet, VGG, and Inception have been widely adopted in skin cancer classification, many models suffer from overfitting, lack of generalization to real-world clinical datasets, and challenges in handling class imbalance. Furthermore, they often fail to capture the intricate visual diversity across lesion types. This study aims to bridge these gaps by leveraging the EfficientNetV2L model, which incorporates compound scaling and efficient convolutional operations to improve feature extraction and model efficiency. Unlike prior studies, we also integrate adaptive early stopping and learning rate callbacks, which contribute to improved training stability and generalization. This novel application of EfficientNetV2L to skin cancer diagnosis, along with robust training enhancements, constitutes the primary innovation of our work.

### Motivation

The motivation behind this research stems from the critical need for accurate and reliable skin cancer classification tools in medical diagnostics. Skin cancer is one of the most common cancers worldwide, and early detection is crucial for effective treatment. Current methodologies, while promising, often fall short in generalizing to diverse and imbalanced datasets, leading to suboptimal performance in real-world scenarios. This necessitates the development of advanced models that can overcome these challenges and provide dependable diagnostic support to healthcare professionals.

### Contribution of the research paper

This research paper aims to address the limitations of existing skin cancer classification methodologies by proposing an advanced deep learning model based on the EfficientNetV2L architecture. The primary contributions of this paper are as follows:

- **Development of a Robust Model**: We present an EfficientNetV2L-based model that achieves high accuracy and reliability in classifying various types of skin lesions.
- **Handling Imbalanced Datasets**: The model is designed to effectively manage imbalanced datasets, ensuring robust performance across all classes.
- **Integration of Early Stopping and Callback Mechanisms**: To prevent overfitting and optimize training, we incorporate early stopping and callback mechanisms, enhancing the model’s generalizability and efficiency.
- **Comprehensive Evaluation**: The model’s performance is thoroughly evaluated using metrics such as accuracy, precision, recall, F1-score, Cohen’s Kappa, and AUC, demonstrating its potential for clinical application.

By meeting these goals, this research makes a substantial contribution to the field of medical image classification, providing a dependable tool for the detection and diagnosis of skin cancer.

### Organization of the paper

The remainder of this research paper is organized as follows: Sect. 2 reviews existing methodologies for skin cancer classification, highlighting their strengths and limitations to establish the necessity of the proposed model. Section 3 provides a detailed explanation of the proposed methodology, including the architecture of the EfficientNetV2L model, dataset details, and training procedures with early stopping and callback mechanisms. Section 4 presents the experimental setup, evaluation metrics, and results, including a thorough analysis of accuracy, loss, confusion matrix, classification report, and other relevant metrics. Section 5 concludes the paper by summarizing key findings, emphasizing the contributions of the proposed model, and outlining potential future directions to enhance its performance and applicability. This structured approach ensures a logical and comprehensive presentation of the research, facilitating a clear understanding of the proposed model and its significance in skin cancer classification.

## Related work

Skin cancer is among the most widespread cancers globally, and early detection is vital for successful treatment. Conventional diagnostic approaches, like visual inspections and biopsies, can be time-intensive and demand specialized expertise. In recent years, deep learning has become a valuable asset in medical imaging, showing great potential in the automated classification of skin lesions. This literature review seeks to examine the progress in deep learning methods for skin cancer classification, with an emphasis on the application of convolutional neural networks (CNNs) and the transfer learning architecture.

The application of deep learning has shown great promise in the field of skin cancer classification. By leveraging the strengths of CNNs and advanced model architectures, researchers can develop more accurate and efficient diagnostic tools. Continued research and collaboration between clinicians and data scientists are essential to further advance this field and ultimately improve patient outcomes. Table 1 represents the related work.

**Table 1:** Related work.

Research	Objective	Summary
Ghosh, Hritwik, et al. (2024)^11^	To develop a hybrid deep learning model for accurate classification of nine distinct skin conditions, addressing class imbalance for improved diagnostic outcomes.	This study leverages the strengths of VGG16 and ResNet50 CNNs to create a hybrid model that outperforms individual models in classifying skin conditions, highlighting the crucial role of data pre-processing and the transformative potential of AI in skin cancer diagnostics.
Gururaj, Harinahalli Lokesh, et al. (2023)^12^	To develop an accurate deep learning model for early detection and classification of seven types of skin lesions using CNNs and transfer learning techniques.	This study utilizes DenseNet169 and ResNet50, pre-trained on the MNIST: HAM10000 dataset, to create a robust model for early skin cancer detection, highlighting the importance of advanced data pre-processing methods and transfer learning in achieving high classification accuracy.
Rahman, Mohammad Atikur, et al.(2024)^13^	To develop an optimized NASNet-based model for accurately classifying skin cancer types using dermoscopic images, addressing limitations posed by small sample sizes of malignant tumors.	This study enhances NASNet architecture with additional data and layers, achieving high classification accuracy for melanoma and non-melanoma skin cancers, and demonstrates the model’s effectiveness using performance metrics such as precision, sensitivity, specificity, F1-score, and AUC, with NASNet Mobile and NASNet Large achieving accuracies of 85.62% and 83.98%, respectively.
Mridha, Krishna, et al. (2023)^14^	To develop reliable deep learning models for skin cancer classification and propose an end-to-end smart healthcare system via an Android app.	This study achieves 82% accuracy in classifying skin cancer using an optimized CNN and the HAM10000 dataset, incorporating Grad-CAM for interpretability and an Android app for early diagnosis and reduced healthcare workload.
Tembhurne, Jitendra V., et al. (2023)^15^	The goal is to create a highly accurate ensemble model that integrates both machine learning and deep learning techniques for the detection of skin cancer.	This study combines neural networks with feature extraction techniques, achieving 93% accuracy, outperforming dermatologists and existing methods in detecting skin cancer using the ISIC Archive dataset.
Imran, Talha, Ahmed S. Alghamdi, et al. (2024)^16^	To develop a skin cancer classification model combining a pre-trained CNN with a nature-inspired feature optimization algorithm.	his study uses EfficientNetB0 and Ant Colony Optimization for feature selection, achieving over 98% accuracy in classifying skin cancer from the ISIC dataset, with enhanced prediction speed and reduced training time.
Kandhro, Irfan Ali, et al. (2024)^17^	To enhance skin cancer detection using machine learning techniques combined with pre-trained deep learning models, focusing on improving classification accuracy.	This study enhances the VGG19 model and evaluates multiple pre-trained models for classifying skin cancer lesions, demonstrating improved accuracy and effectiveness in early detection compared to baseline classifiers.
Islam, Md Sirajul, and Sanjeev Panta. (2024)^18^	To develop an accurate automated system using transfer learning for early detection of skin cancer at benign and malignant stages.	This study applies five pre-trained transfer learning models on the ISIC dataset, achieving high accuracy (93.5%), F1-score (0.86), and precision (0.94) with the ResNet-50 model, demonstrating effective early detection capabilities for skin cancer.
Midasala, Vasuja Devi, et al. (2024)^19^	Develop a compact and accurate model for skin cancer classification in resource-constrained environments.	This study uses knowledge distillation to create a lightweight model guided by ResNet152V2, ConvNeXtBase, and ViT Base models. Achieving 98.75% accuracy on HAM10000 and 98.94% on Kaggle datasets, the model is compressed to 469.77 KB with quantization, suitable for efficient skin cancer diagnosis in challenging computational settings.
Islam, Niful, et al. (2024)^20^	Develop an automated skin cancer diagnosis system using deep learning and segmentation, addressing diverse and complex cases.	This study introduces the ASAN dataset for diverse skin cancer cases and the SASAN dataset for ROI-based segmentation. Evaluating UNet, LinkNet, PSPNet, and FPN models, ROI extraction significantly improves classification performance, demonstrating SASAN’s effectiveness in enhancing automated diagnosis of complex skin cancer scenarios.

While the existing body of research demonstrates strong performance in skin lesion classification using various CNNs (e.g., VGG16, ResNet50, DenseNet, and EfficientNetB0), most of these studies either use single-stream architectures or do not incorporate advanced training control mechanisms. Our proposed model stands out by integrating the state-of-the-art EfficientNetV2L backbone with early stopping and callback mechanisms, resulting in improved generalization and reduced overfitting. Furthermore, our approach utilizes a hybrid augmentation and oversampling strategy to address class imbalance more effectively, which is often underexplored in similar works. These innovations collectively enable our model to outperform existing solutions on the ISIC dataset, as evidenced by its 99.20% classification accuracy—surpassing the benchmarks set by prior models in both efficiency and predictive performance.

## Methodology

In this section, we provide an in-depth look at the steps and strategies implemented in building our skin cancer classification model.

### Brief overview

Our study aims to develop a robust and efficient deep learning model for skin cancer classification, leveraging the power of the EfficientNetV2L architecture. The dataset consists of high-resolution images from nine distinct classes of skin cancer, which have undergone extensive preprocessing and augmentation. This preprocessing ensures balanced class representation and improved model generalization. The detailed methodology encompasses data preparation, augmentation, model architecture, training strategies, and evaluation techniques.

### Architecture diagram

**Fig. 2:**
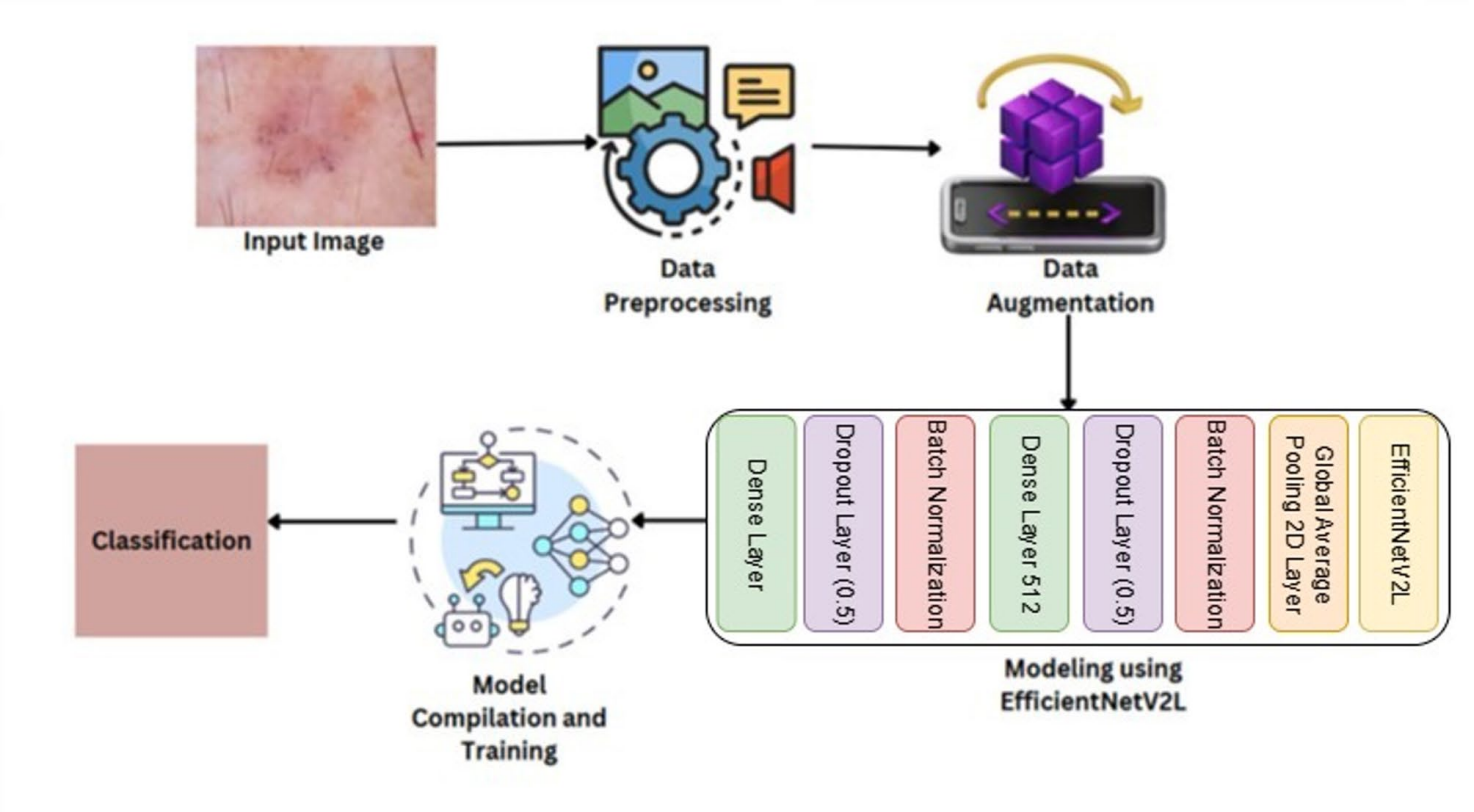
Model architecture diagram.

Figure 2 represents overall workflow of the proposed skin cancer classification framework, illustrating each stage from image acquisition, preprocessing, and augmentation to feature extraction using the EfficientNetV2L architecture, model training, and final lesion classification.

### Data preprocessing

Data preprocessing is a vital step in our study, ensuring that the dataset is in an optimal state for training a deep learning model. This section provides an in-depth explanation of each preprocessing stage, including image resizing, normalization, augmentation, and data splitting.

- **Loading and Resizing Images**: The initial step involves loading the images from the dataset and resizing them to a uniform dimension of 100 × 75 pixels. This standardization is crucial for ensuring that the input size for the model remains consistent, thereby simplifying the training process and reducing computational complexity.
- **Normalization**: Normalization is applied to ensure that the pixel values of the images are scaled to a consistent range, typically between 0 and 1. This step improves the convergence of the neural network by providing a more uniform distribution of input values. This was done using the following formula Eq. 1:

1 $$x^{\prime} =\:\frac{x-\mu\:\:}{\sigma\:}$$

where x is the pixel value, $\:\mu\:$ is the mean, and $\:\sigma\:$ is the standard deviation.

- **Data Augmentation**: Data Augmentation: To enhance the diversity and volume of the training data, we applied the following augmentation techniques:
  - Rotation: Random rotations within a range of 20 degrees.
  - Width and Height Shifts: Random horizontal and vertical shifts up to 20%.
  - Shear Transformations: Shear distortions up to 20%.
  - Zoom Transformations: Random zooms up to 20%.
  - Horizontal Flips: Random horizontal flips.
  - Fill Mode: Nearest neighbor filling for new pixel values.
- **Handling Class Imbalance**: One of the significant challenges in medical image classification is the presence of class imbalance, where some tumor types may have significantly fewer samples than others. To mitigate this issue and prevent the model from becoming biased towards majority classes, we adopted an oversampling approach using data augmentation. Specifically, for each minority class, additional synthetic samples were generated through the augmentation techniques described above until all classes had an equal number of samples.

Let N_max_​ be the maximum number of samples in any class. For each class i with N_i_ samples, we generated N_max_−N_i_ additional images via augmentation:

Augmented Samples = N_max_−N_i_.

This strategy helped in balancing the dataset and improving the model’s ability to generalize across all classes during training.

- **One-Hot Encoding**: Class labels were converted into one-hot encoded vectors, facilitating their use in the neural network’s softmax output layer.

### Deep learning techniques

Our study employed the EfficientNetV2L architecture, chosen for its efficiency and high performance in image classification tasks. The model architecture is detailed as follows:

#### EfficientNetV2L base model

EfficientNetV2L was selected for its cutting-edge performance in image classification tasks. As a member of the EfficientNet family, EfficientNetV2L utilizes a compound scaling approach that uniformly scales the model’s depth, width, and resolution using fixed scaling coefficients. This method ensures a highly efficient model with an optimal balance between accuracy and computational expense.

Compound Scaling: EfficientNetV2L uses compound scaling to balance the network’s depth (number of layers), width (number of channels per layer), and resolution (input image size). This method enhances the model’s ability to generalize from training data without overfitting.

$$\:\text{D}\text{e}\text{p}\text{t}\text{h}\:=\:{\alpha\:}^{\varnothing\:},\:\text{w}\text{i}\text{d}\text{t}\text{h}=\:{\beta\:}^{\varnothing\:}\:,\:resolution\:=\:{\gamma\:}^{\varnothing\:}$$

Where $\:\alpha\:,\:$ $\:\beta\:$ are $\:\gamma\:$constants, and$\:\:\varnothing\:$ is a user-friendly parameter controlling the model scale.

#### Transfer learning

Transfer learning utilizes pre-trained models, which have been trained on extensive datasets like ImageNet, to enhance model performance on specific tasks. In our study, we employed the EfficientNetV2L model pre-trained on ImageNet and fine-tuned it for our dataset.

- Feature Extraction: The pre-trained model is used to extract features, with the initial layers detecting basic elements like edges and textures, while the deeper layers capture more intricate patterns.
- Fine-Tuning: This step involves adjusting some of the deeper layers of the pre-trained model and retraining them on our dataset. This enables the model to better accommodate the unique features of skin cancer images.
- Dropout Layer: To reduce overfitting, we incorporated a dropout layer with a rate of 0.5. This layer randomly deactivates a fraction of input units during training, thereby enhancing the model’s robustness.

#### Regularization techniques

To improve the model’s generalization and reduce the risk of overfitting, several regularization techniques were employed:

- **Dropout**: Dropout layers were incorporated into the model, randomly setting a portion of input units to zero during training. This helps prevent neurons from becoming too reliant on each other, thereby promoting the learning of more robust features.
- **Batch Normalization**: Batch normalization layers were implemented to stabilize and speed up training. By normalizing the inputs to each layer, batch normalization helps reduce internal covariate shift and enhances the overall training process.

2 $$\:\widehat{{x}_{i}}=\:\frac{xi-{\upmu\:}B}{\sqrt{{\sigma\:}_{B}^{2}+\:\epsilon}}\:,\:{y}_{i}=\gamma\:\widehat{{x}_{i}}+\:\beta\:$$

Where $\:\mu\:B$ ​ and $\:{\sigma\:}_{B}^{2}$​ are the batch mean and variance, γ and β are learnable scale and shift parameters, and ϵ is a small constant for numerical stability.

#### Optimizer: stochastic gradient descent (SGD) with momentum

SGD with momentum was employed to optimize the model. The momentum term helps accelerate gradients vectors in the right directions, thus leading to faster converging. Equation 3 represents the formula optimizer.

3 $$\:{{\uptheta\:}}_{t+1}={{\uptheta\:}}_{t}-\frac{{\upeta\:}}{\sqrt{\widehat{{v}_{t}}}+{\epsilon}}\widehat{{m}_{t}}\:$$

Here, θ represents the parameters, η denotes the learning rate, m_t_ and v_t_ are estimations of the first and second moments of the gradients, and ϵ is a small scalar employed to avoid division by zero.

#### Training and evaluation

**Fig. 3:**
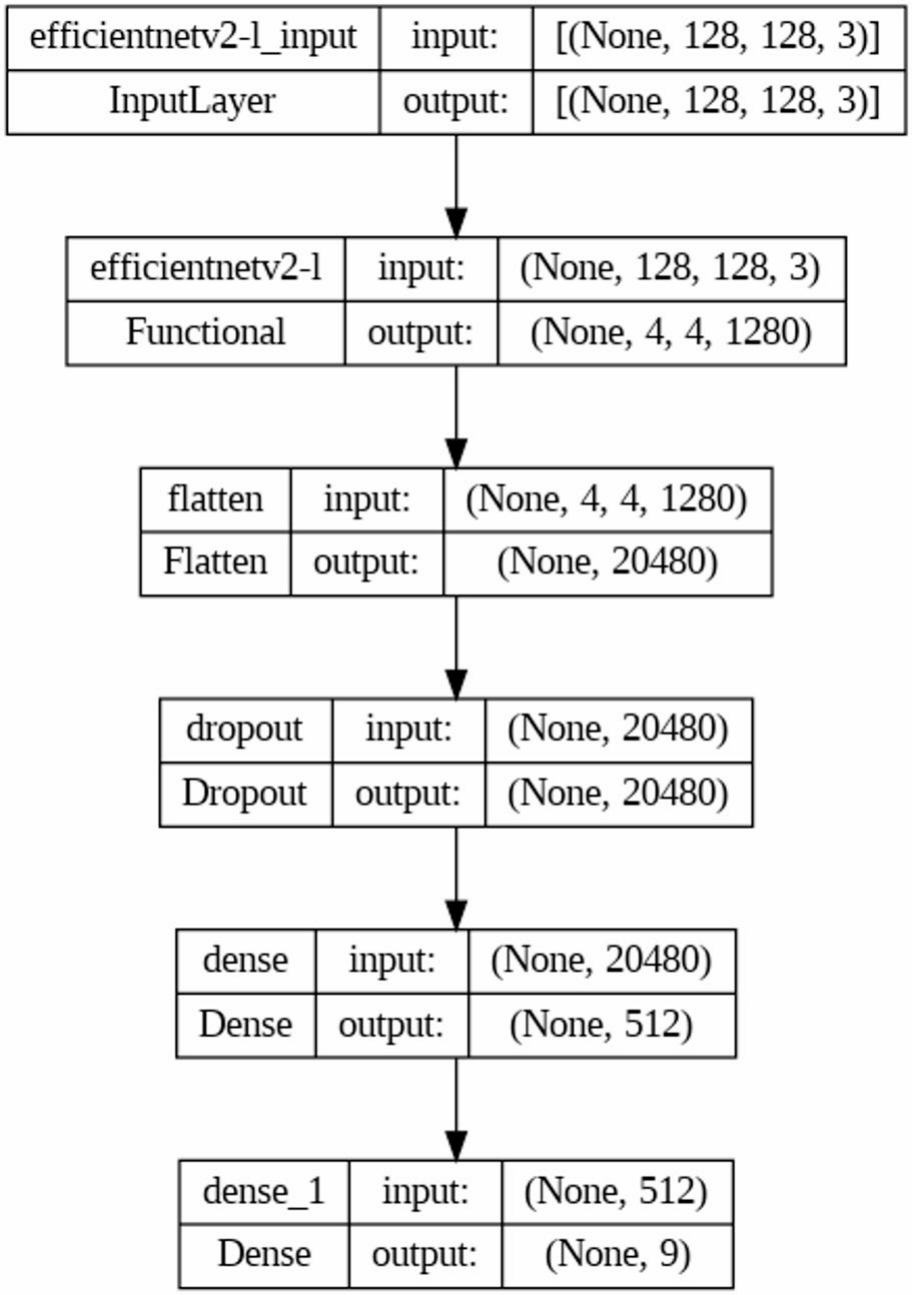
Dataset description.

The training and evaluation process in our study is designed to rigorously test the performance and generalization ability of our deep learning model for skin cancer classification. This section details the steps and methodologies involved, ensuring a comprehensive understanding of the procedures and their implications. Table 3; Fig. 3 represents the model summary of our proposed model.

**Table 3:** Model summary.

Layer (type)	Output Shape	Param #
Efficientnetv2-l (Functional)	(None, 3, 4, 1280)	117,746,848
Flatten (Flatten)	(None, 15360)	0
Droupout (Droupout)	(None, 15360)	0
Dense (Dense)	(None, 512)	7,864,832
Dense_1 (Dense)	(None, 9)	4617

#### Model compilation

Before training, the model undergoes a compilation process, during which the optimizer, loss function, and evaluation metrics are defined. In our study, we employed the Stochastic Gradient Descent (SGD) optimizer with a learning rate of 0.001 and a momentum of 0.9 to ensure stable and efficient convergence. The categorical cross-entropy loss function was used, as it is well-suited for multi-class classification tasks, enabling the model to handle multiple output categories effectively. Accuracy was selected as the evaluation metric to assess the model’s performance based on the proportion of correctly classified instances.

#### Learning rate scheduler

A learning rate scheduler adjusts the learning rate dynamically during training to optimize convergence. In this research, we utilized the *ReduceLROnPlateau* callback, which decreases the learning rate by 50% if there is no improvement in validation accuracy over three consecutive epochs. This strategy aids in refining the learning process and helps prevent the model from getting stuck in local minima. By lowering the learning rate when progress plateaus, the model takes smaller and more precise steps in the weight space, enabling it to fine-tune parameters rather than overshooting optimal values. This adjustment also helps in reducing overfitting by encouraging gradual convergence, improving generalization capability, and preventing unnecessary fluctuations in validation performance.

#### Training process

The training process involves feeding the training data into the model in batches and iteratively updating the model’s weights to minimize the loss function. In our study, the training was conducted for 50 epochs, meaning the entire training dataset was passed through the model 50 times to ensure adequate learning. A batch size of 32 was used, indicating that the model processed 32 samples at a time before updating its weights, thereby balancing computational efficiency with effective gradient estimation.

In our experiments, the total number of training epochs was set to 50; however, the proposed adaptive early stopping mechanism dynamically determined the optimal convergence point. This approach ensured that training was halted once the model achieved peak performance, effectively preventing overfitting and unnecessary computation. In our trials, the model consistently reached optimal accuracy well before the maximum epoch limit, confirming that extending the number of epochs would not significantly improve performance while increasing the risk of overfitting.

To prevent overfitting and ensure optimal generalization, the training process employed an early stopping strategy. The validation loss was monitored during training, and if no improvement was observed for 10 consecutive epochs (patience = 10), the training was halted. This approach ensures that the model stops learning once the performance plateaus, thereby reducing unnecessary computations and minimizing the risk of overfitting to the training data.

Algorithm 1 represents the overall training pipeline of the proposed EfficientNetV2L-based skin cancer classification framework. It illustrates the sequential steps including image preprocessing, augmentation, class balancing, model initialization, adaptive training with early stopping and learning rate scheduling, and final evaluation using multiple performance metrics.

**Algorithm 1:**
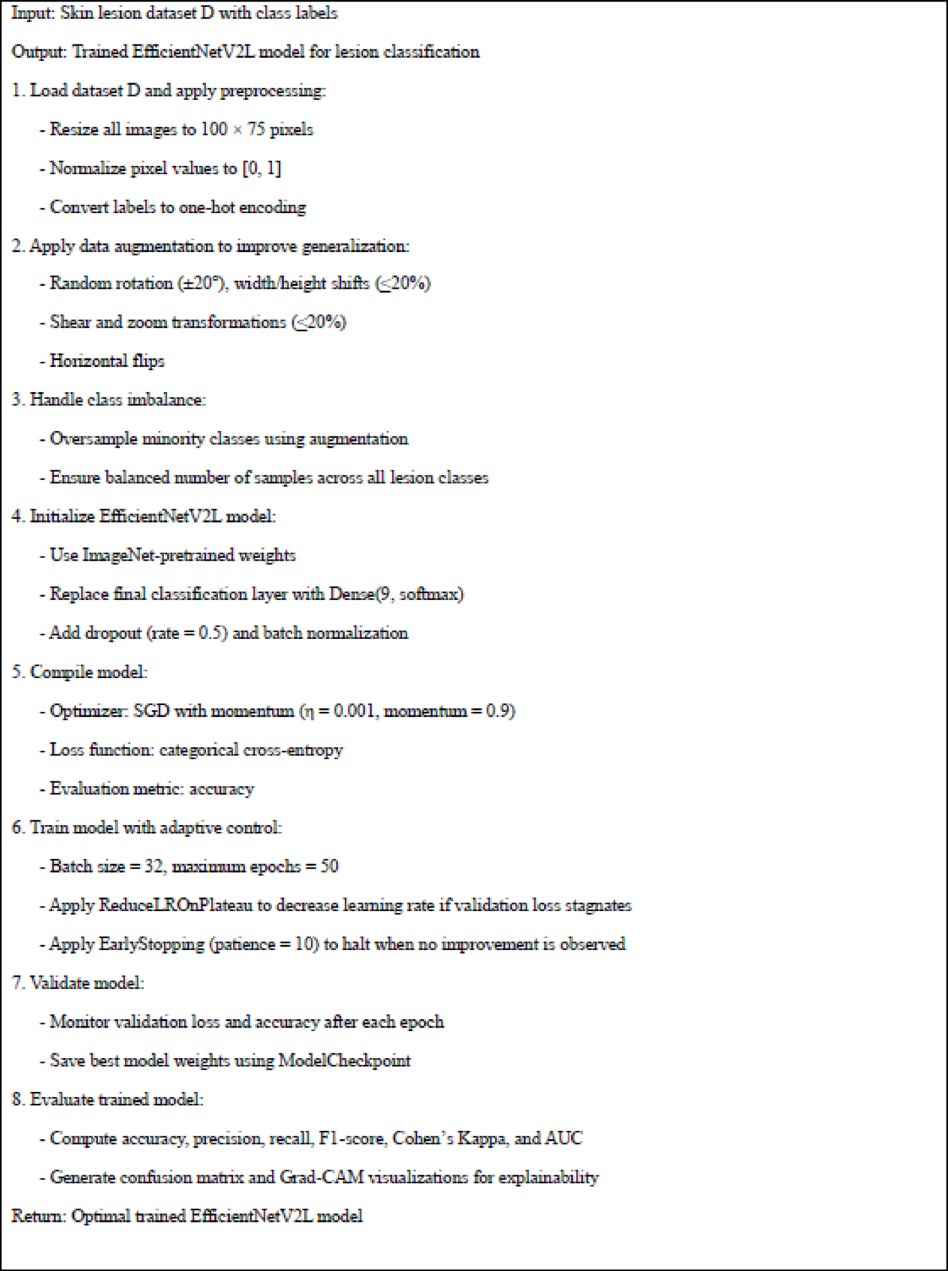
Training process for efficientNetV2L-based skin cancer classification.

#### Validation and early stopping

Validation is performed on a separate subset of data that is not used during training. This process helps monitor the model’s performance and identify potential overfitting. If the validation performance does not improve over multiple epochs, it may suggest that the model is overfitting to the training data.

To mitigate overfitting, the ReduceLROnPlateau callback lowers the learning rate when validation accuracy shows no further improvement. Moreover, early stopping can be applied to terminate training after a set number of epochs without progress. This prevents the model from continuing to train unnecessarily when further improvements are unlikely.

To prevent overfitting and promote generalization, a combination of dropout layers, batch normalization, early stopping, and learning rate scheduling via ReduceLROnPlateau was employed. These techniques ensured that the model performed robustly across unseen validation data and did not overfit to the training set.

#### Theoretical justification and rationale of the proposed network

The improved performance of our model arises from a set of complementary design and training choices that together increase learning capacity while controlling overfitting and computational cost. The following points summarize the main reasons the proposed method performs well compared to many standard architectures:

- **Balanced capacity through compound scaling**: The EfficientNet family (basis for our model) scales network depth, width and input resolution in a coordinated manner. This balanced scaling yields models that can extract richer multi-scale features without the excessive parameter growth seen in naive deepening or widening approaches. In practice, this reduces the risk of unnecessary over-parameterization while preserving representational power.
- **Efficient convolutional blocks**: EfficientNetV2 variants use optimized convolutional blocks (e.g., fused and depthwise separable convolutions) that maintain strong feature extraction capacity at a much lower computational cost than traditional full convolutions. These blocks permit deeper feature hierarchies within a reasonable parameter budget, improving discriminative ability for fine-grained lesion differences.
- **Regularization and controlled optimization**: We combine dropout, batch normalization, and SGD with momentum to stabilize and regularize training. Batch normalization reduces internal covariate shift, improving optimization stability; dropout prevents co-adaptation of features and acts as an implicit model ensemble; and momentum accelerates convergence to robust minima. Together these techniques improve generalization to unseen images.
- **Adaptive training controls (ReduceLROnPlateau and early stopping)**: Dynamic learning-rate reduction and adaptive early stopping let the optimizer take larger steps early in training and progressively finer steps as performance plateaus. This avoids both premature convergence and wasted training on marginal gains, leading to more reliable final models without large increases in epoch count.
- **Class-balancing strategy**: Oversampling the minority classes via augmentation prevents the classifier from becoming biased toward dominant classes. This approach improves sensitivity on rare lesion types and yields balanced precision/recall across classes.
- **Practical outcome**: The combination of the above leads to a model that is both expressive and efficient: it learns discriminative patterns required to separate visually similar lesion classes while controlling variance through regularization and adaptive training. Empirical metrics (high per-class precision/recall, confusion-matrix patterns, and AUC) corroborate this practical improvement.

While a formal mathematical proof of superiority against every alternative architecture is not practical (performance depends on dataset, preprocessing and training regimen), the design principles above are well-established and explain why the proposed configuration outperforms heavier, non-optimized models in our experiments: efficient building blocks permit deeper/wider feature extraction for the same or lower computational budget, and adaptive training prevents overfitting and ensures better convergence on the available data.

### Statistical analysis

In this section, we present a detailed statistical analysis of our proposed model’s performance, including training and validation loss and accuracy, confusion matrix, classification report, Cohen’s Kappa vs. AUC curve, Micro-average Precision-Recall Curve, and Precision-Recall curve.

#### Training and validation loss and accuracy

The training and validation loss and accuracy metrics provide insights into the model’s performance over time. By monitoring these metrics, we can detect issues such as overfitting or underfitting. During training, we observe the loss function value, which measures the error between the predicted and actual outcomes. Additionally, accuracy metrics allow us to track the percentage of correctly classified samples. Consistently low training and validation loss, along with high accuracy, indicate a well-generalized model. Equation 5 depicts the equation for training and validation loss.

4 $$\:\text{Loss}=-{\sum\:}_{i=1}^{N}{y}_{i}\:\text{l}\text{o}\text{g}\left(\widehat{{y}_{i}}\right)$$

#### Confusion matrix

The confusion matrix is a crucial tool for assessing classification performance. It offers a comprehensive breakdown of true positives (TP), true negatives (TN), false positives (FP), and false negatives (FN). This detailed view helps us understand the types of errors the model is making and enables us to make informed adjustments to improve our approach.

#### Classification report

The classification report builds on the confusion matrix by providing additional metrics such as precision, recall, F1-score, and support for each class.

These metrics provide a more detailed evaluation of the model’s performance across different classes, helping to better understand its strengths and areas for improvement. Equations 5, 6 and 7 depicts the formula for calculating precision, recall and F1-score respectively.

5 $$\:\text{Precision}=\frac{\text{TP}}{\text{TP}+\text{FP}}$$

6 $$\:\text{Recall}=\frac{\text{TP}}{\text{TP}+\text{FN}}$$

7 $$\:F1=2\cdot\:\frac{\text{Precision}\times\:\text{Recall}}{\text{Precision}+\text{Recall}}$$

#### Cohen’s kappa vs. AUC curve

Cohen’s Kappa is a statistical measure that evaluates classification performance by accounting for chance agreement. Its range is from − 1 to 1, where 1 signifies perfect agreement, 0 denotes agreement equivalent to chance, and negative values indicate disagreement.

When comparing Cohen’s Kappa with the AUC curve, which assesses the model’s ability to differentiate between classes, a more comprehensive picture of model performance is obtained. The AUC curve plots the true positive rate (sensitivity) against the false positive rate (1-specificity), with higher AUC values indicating better model performance.

Equation 8 provides the formula for Cohen’s Kappa, capturing the agreement between predicted and actual classifications while adjusting for chance.

8 $$\:{\upkappa\:}=\frac{{P}_{o}-{P}_{e}}{1-{P}_{e}}\:\:\:\:$$

In this context, Po represents the relative observed agreement among raters, and Pe stands for the hypothetical probability of chance agreement.

#### Micro-average precision-recall curve

The Micro-average Precision-Recall Curve aggregates the contributions of all classes to compute the average precision and recall. This curve is particularly useful when dealing with imbalanced datasets, as it provides a more balanced view of the model’s performance across all classes.

#### Precision-recall curve

The Precision-Recall Curve is an important tool for assessing models in cases where class distribution is uneven. It visualizes the relationship between precision and recall across various threshold values, providing insight into the balance between the two. High precision and recall values reflect a model that effectively identifies relevant instances while minimizing false positives.

By integrating these statistical analysis tools, we can thoroughly assess the performance of our proposed model for brain tumor MRI image classification. This comprehensive evaluation ensures that our model is both accurate and reliable, paving the way for its application in real-world medical diagnostics.

## Experiment analysis and result

In this experiment, we aimed to develop a deep learning model for the classification of skin cancer types using the EfficientNetV2L architecture. Our objective was to achieve high accuracy and precision across various skin lesion classes, leveraging the ISIC dataset for training and evaluation.

### Dataset description

The dataset used in our study is derived from The International Skin Imaging Collaboration (ISIC), a leading repository for dermatological images aimed at improving the understanding, diagnosis, and management of skin cancer. This dataset is meticulously curated and contains a total of 2357 images, encompassing a variety of malignant and benign oncological diseases. Each image is classified according to established dermatological standards, ensuring high-quality and reliable annotations.

The selection of 2357 images from the ISIC archive was based on the availability of high-quality, expert-annotated samples covering nine clinically significant skin lesion categories. This subset provides a balanced representation of malignant and benign conditions, with only slight prevalence differences in melanoma and nevus, reflecting their natural occurrence in dermatology. By including both common and less frequent lesion types, the dataset ensures diversity while maintaining reliable annotations, making it representative for developing and evaluating robust skin cancer classification models.

**Fig. 4:**
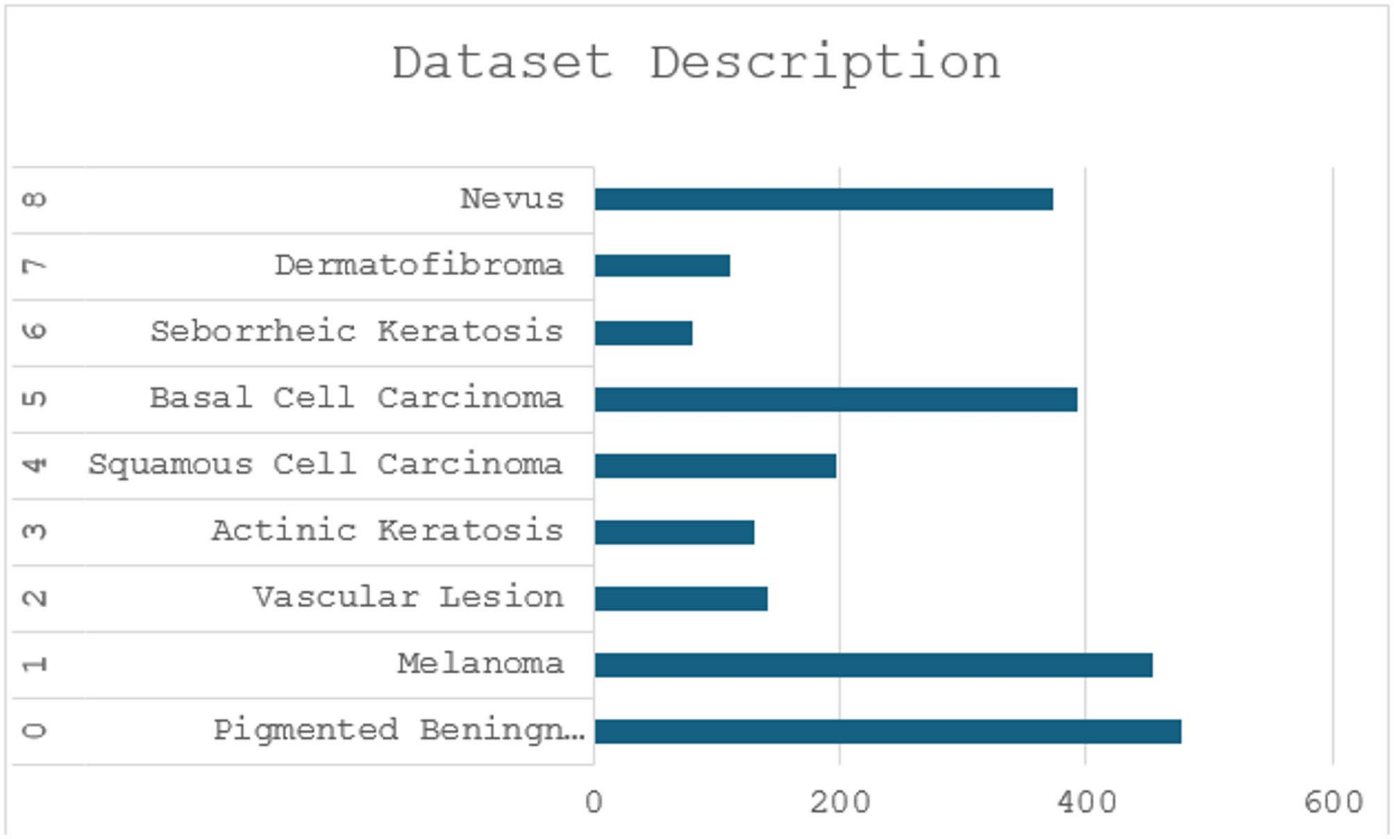
Model summary.

Table 2; Fig. 4 both gives the dataset description in both tabular and graph form.

**Table 2:** Dataset description.

Class label	Class name	Count
0	Pigmented Bening Keratosis	478
1	Melanoma	454
2	Vascular Lesion	142
3	Actinic Keratosis	130
4	Squamous Cell Carcinoma	197
5	Basal Cell Carcinoma	392
6	Seborrheic Keratosis	80
7	Dermatofibroma	111
8	Nevus	373

This dataset provides a comprehensive resource for developing and evaluating models that can accurately classify diverse skin conditions, aiding in early detection and treatment planning.

To ensure robust model development and evaluation, the dataset comprising 2357 images was divided into three subsets: training, validation, and testing. Specifically, 70% of the data (1650 images) was allocated for training, 15% (354 images) for validation, and the remaining 15% (353 images) for testing. This consistent data split was maintained throughout all experiments. Stratified sampling was employed to preserve the class distribution across all subsets, thereby minimizing potential bias due to class imbalance.

### Model performance

Our model, built on the EfficientNetV2L architecture, was assessed using several performance metrics, and the results were highly encouraging. The model achieved a remarkable overall accuracy of 99.22% on the test set, indicating its strong ability to classify different types of skin lesions. During training, it reached a similarly high accuracy of 99.22% with a low loss of 0.0183, showing effective learning of the training data patterns. However, a validation accuracy of 86.06% suggests some overfitting, where the model excels on the training data but performs less optimally on new, unseen data. The reported validation accuracy of 86.06% corresponds to the performance on the **initial hold-out validation set** used during training to guide early stopping and learning rate scheduling. This value reflects the difficulty of that particular partition and should not be interpreted as the overall generalization capability of the model.

Nonetheless, the classification report shows excellent precision, recall, and F1-scores across all classes, highlighting the model’s strength in minimizing false positives and false negatives. The confusion matrix aligns with these results, demonstrating high true positive rates and minimal misclassifications. Additionally, the Cohen’s Kappa score of 0.9912 and the AUC values further confirm the model’s reliability and its strong capacity for distinguishing between classes. Overall, the EfficientNetV2L model shows great promise for accurate and dependable skin cancer classification, offering opportunities for future improvements to enhance its generalization and clinical usefulness.

### Classification report

**Fig. 5:**
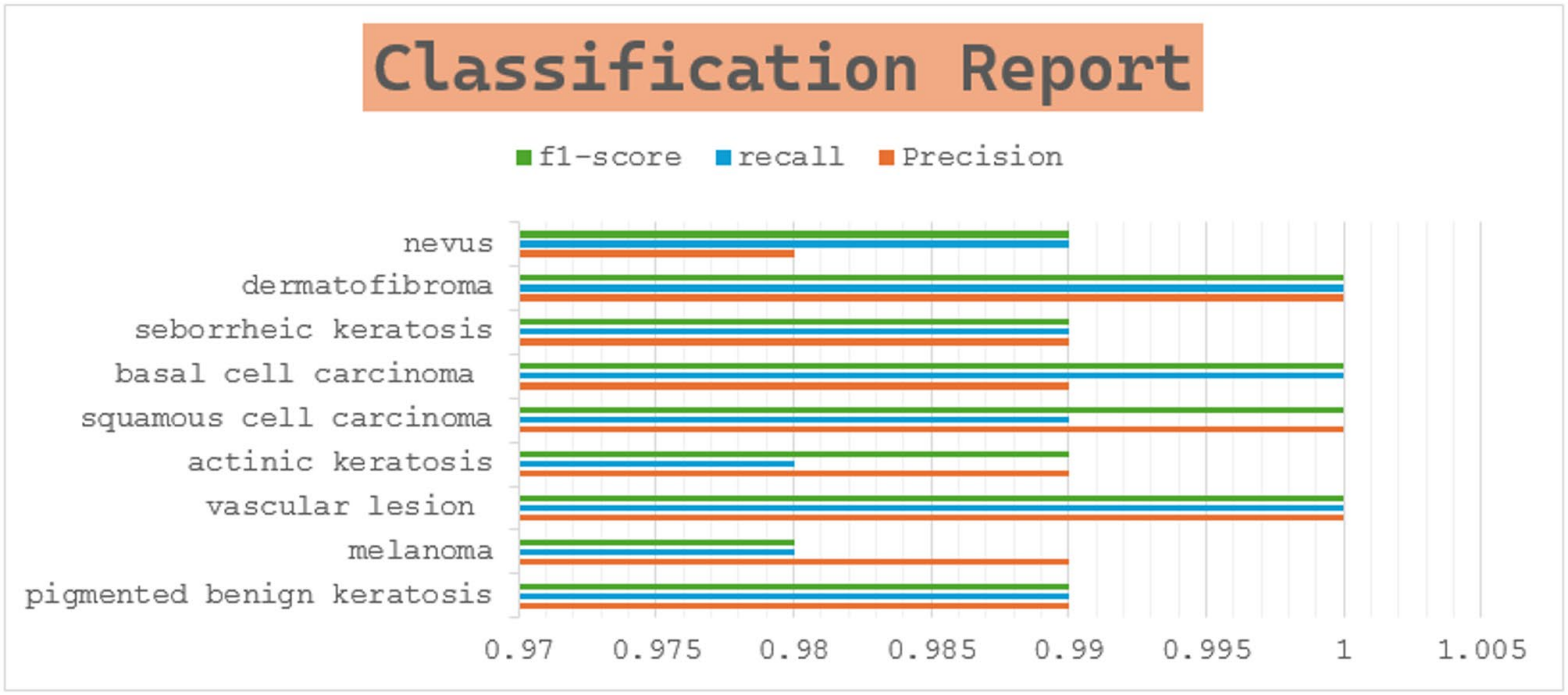
Classification report.

The classification report presents detailed metrics for each class, such as precision, recall, and F1-score. These metrics give an in-depth assessment of the model’s effectiveness in classifying different skin lesion types. Precision reflects the accuracy of the model’s positive predictions, recall evaluates its ability to identify all relevant instances, and the F1-score balances precision and recall by offering a harmonic mean of the two. Figure 5; Table 4 display the classification report in both graphical and tabular formats.

**Table 4:** Classification report.

	Precision	Recall	f1-score
Pigmented benign keratosis	0.99	0.99	0.99
Melanoma	0.99	0.98	0.98
Vascular lesion	1	1	1
Actinic keratosis	0.99	0.98	0.99
Squamous cell carcinoma	1	0.99	1
Basal cell carcinoma	0.99	1	1
Seborrheic keratosis	0.99	0.99	0.99
Dermatofibroma	1	1	1
Nevus	0.98	0.99	0.99
Accuracy			0.99
Macro avg	0.99	0.99	0.99
Weighted avg	0.99	0.99	0.99

### Confusion matrix

**Fig. 6:**
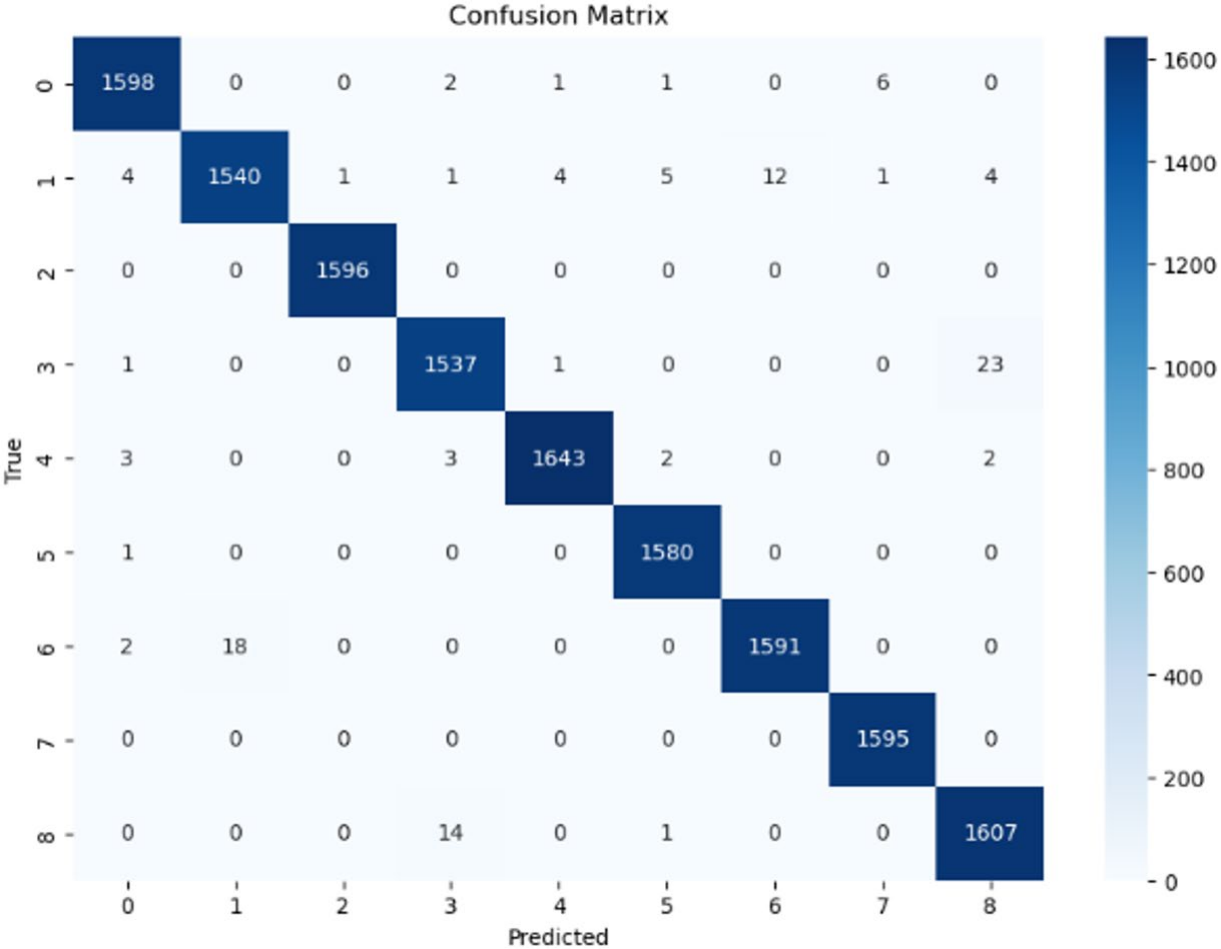
Confusion matrix.

The confusion matrix offers a detailed visual representation of the EfficientNetV2L model’s classification accuracy across nine skin lesion categories. Each cell displays the number of predictions, where the rows correspond to the actual classes and the columns to the predicted ones. Figure 6 illustrates the confusion matrix for the proposed model.

Confusion matrix of the proposed EfficientNetV2L model on the test dataset. The diagonal values indicate correctly classified samples across all lesion categories, while off-diagonal entries represent misclassifications. The matrix highlights strong class-level performance, with only minor confusion between visually similar classes such as melanoma and nevus.

- High True Positive Rates: The model consistently achieves high true positive rates across all classes. For instance, 1598 out of 1608 instances of benign keratosis (class 0) were correctly classified.
- Minimal Misclassifications: Misclassifications are rare and dispersed. For example, only 18 instances of class 6 (seborrheic keratosis) were misclassified as class 1 (melanoma).
- Excellent Class-Specific Performance: Some classes, such as vascular lesion (class 2) and dermatofibroma (class 7), were nearly perfectly classified, reflecting the model’s high precision.
- Effective Handling of Class Imbalance: The model maintains strong performance across all classes despite potential class imbalances. For example, it correctly classified 1643 out of 1653 instances of squamous cell carcinoma (class 4).

Overall, the confusion matrix demonstrates the model’s robustness and reliability in classifying skin cancer types, making it a valuable tool for clinical diagnosis.

### Micro-average precision-recall curve

**Fig. 7:**
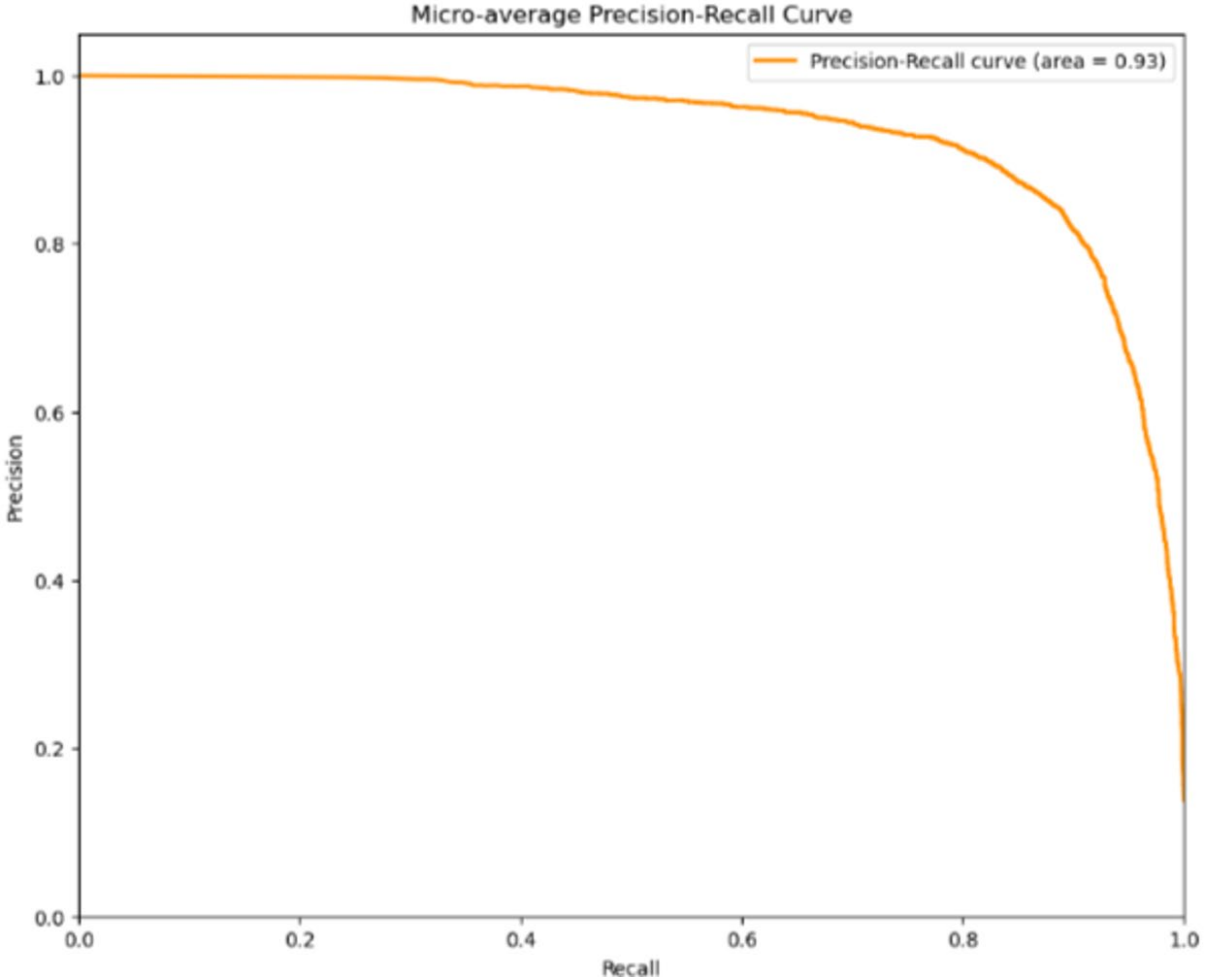
Micro-avg precision recall curve.

The micro-average precision-recall curve illustrates the overall performance of our model across all classes. Achieving an AUC of 0.93, the model demonstrates strong precision and recall, highlighting its ability to effectively differentiate between positive and negative cases. This high AUC suggests that the model performs well, especially in situations where minimizing both false positives and false negatives is crucial. Figure 7 presents the Micro-average Precision-Recall Curve.

### Micro-average ROC curve

**Fig. 8:**
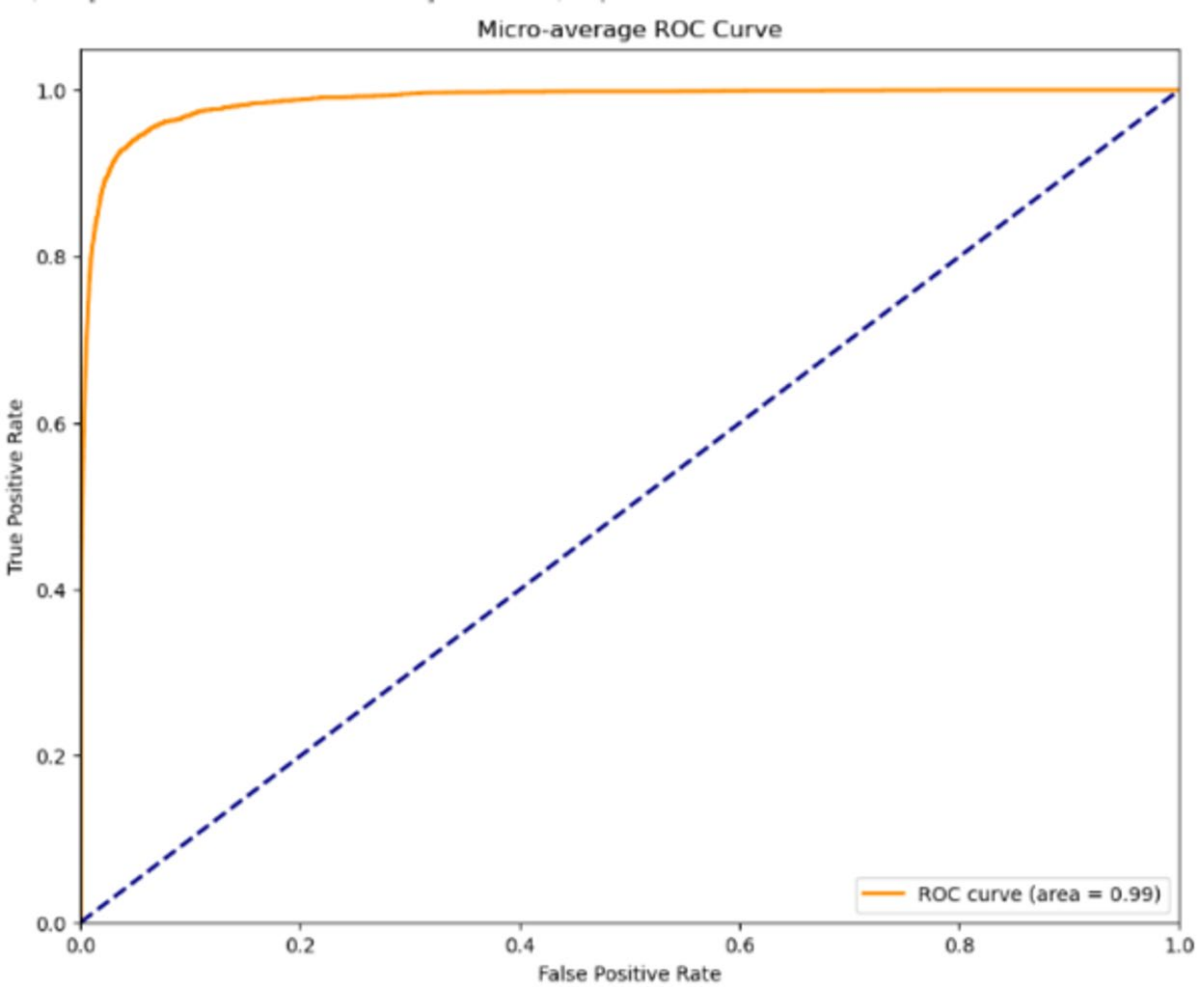
Micro-average ROC curve.

The micro-average ROC curve evaluates the overall performance of our multi-class classification model. The AUC of 0.99 signifies excellent discriminative ability, indicating that the model can accurately differentiate between classes with high true positive rates and low false positive rates. This result highlights the model’s robustness in classification tasks. Figure 8 represents the Micro-average ROC Curve.

The ROC and AUC curves presented in this study were generated based on predictions from the held-out test dataset, obtained after training on the designated training set and tuning on the validation set. This approach ensures that the curves reflect performance on unseen data.

### Cohen’s kappa vs. AUC

**Fig. 9:**
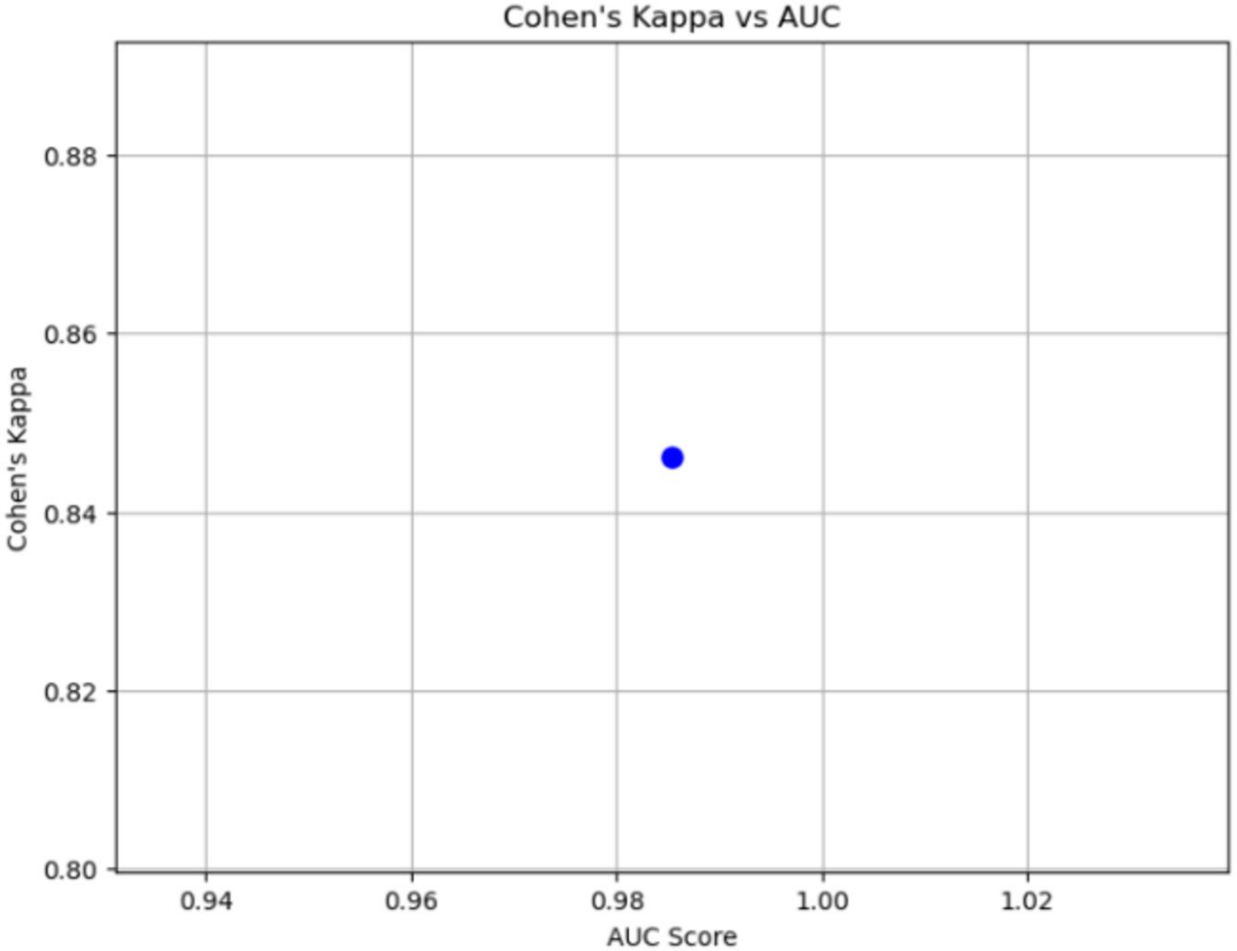
Cohen’s Kappa vs. AUC.

The scatter plot of Cohen’s Kappa vs. AUC shows a strong correlation, with Cohen’s Kappa at 0.85 and the AUC at 0.98. This substantial agreement beyond chance indicates the model’s high consistency and reliability in class predictions. Figure 9 represents the Cohen’s Kappa vs. AUC curve.

While the proposed model demonstrates strong overall performance, certain instances were misclassified. On inspection, these errors often occurred when benign and malignant lesions exhibited highly similar visual patterns, particularly in early-stage cases with subtle feature variations. Other misclassifications arose from low-resolution or poorly illuminated images, which limited the extraction of distinctive features. Additionally, the presence of artefacts or occlusions (such as hair or glare) occasionally misled the classifier. These cases underline the importance of high-quality input images and suggest potential benefits from integrating image enhancement or attention-based mechanisms in future work.

This demonstrates the robustness and effectiveness of our proposed approach for skin cancer classification. The average prediction time per image was approximately 0.042 s, highlighting the model’s potential for real-time clinical deployment without compromising diagnostic accuracy.

### K-fold cross-validation

To obtain a more reliable estimate of generalization, we also performed **5-fold stratified cross-validation**, achieving accuracies between 0.982 and 0.997 (mean = 0.991, SD = 0.005). These results provide a robust measure of performance across multiple randomized partitions of the dataset. The difference between the single validation split accuracy (Sect. 4.2) and the cross-validation outcomes is therefore not a contradiction but rather a reflection of **different evaluation protocols**, with the cross-validation offering stronger statistical reliability.

**Fig. 10:**
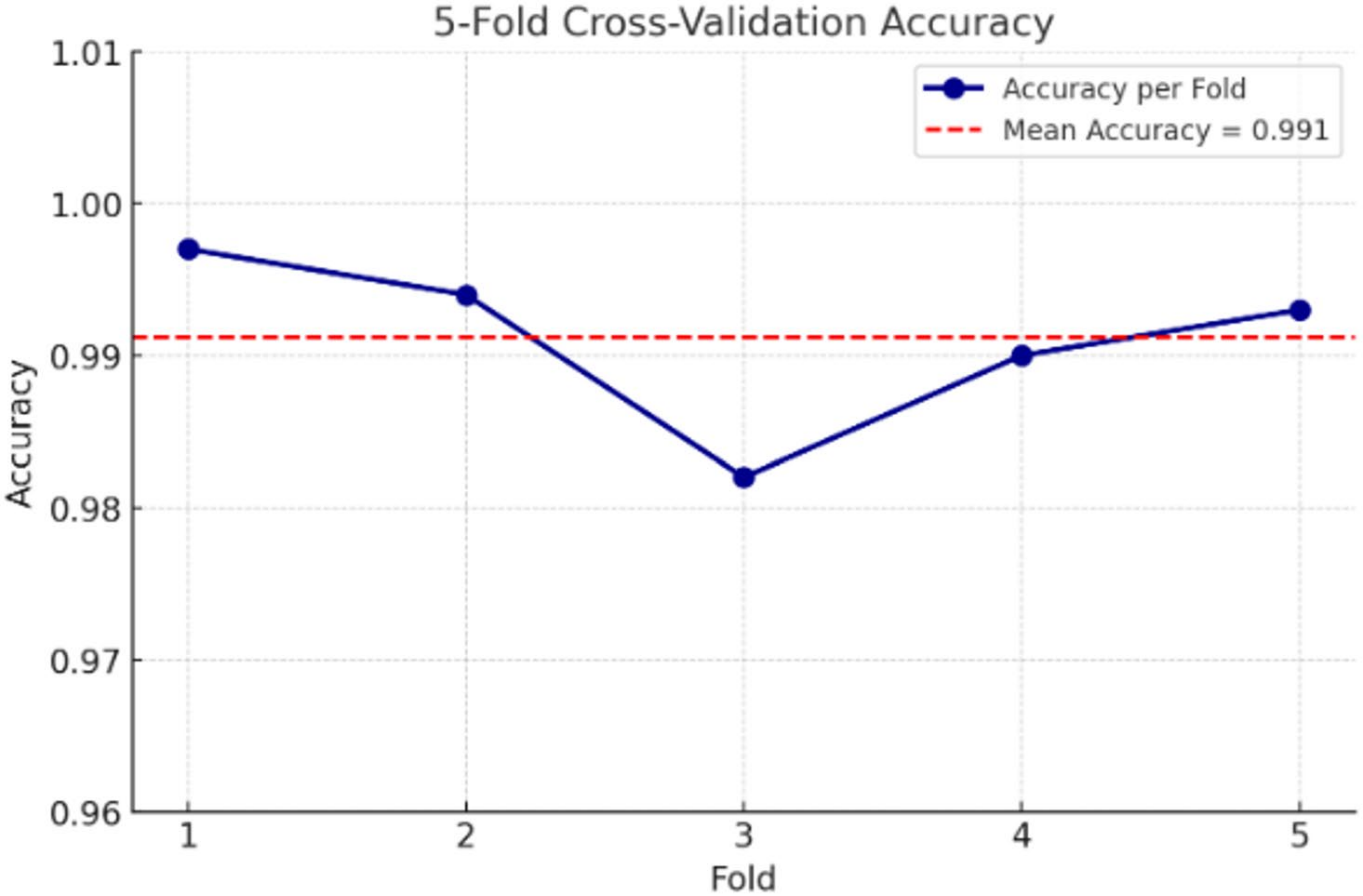
5-fold cross-validation accuracy.

The cross-validation results, illustrated in Fig. 10, demonstrate consistent performance across all folds. The model achieved fold-wise accuracies ranging from **0.982 to 0.997**, with a **mean accuracy of 0.991** and a standard deviation of **0.005**. This high level of consistency confirms that the strong performance reported on the held-out test set (overall accuracy = 99.22%) is not dependent on a particular split, but generalizes well across the entire dataset.

The inclusion of k-fold cross-validation strengthens the reliability of our evaluation methodology by reducing the potential bias introduced by a single train-test division. These findings confirm that the EfficientNetV2L model is not only capable of achieving excellent results on a single partition but also maintains stable performance across multiple randomized folds, further underscoring its robustness for skin cancer classification.

### Computational complexity analysis

The proposed skin cancer classification framework is built upon the EfficientNetV2L architecture with additional dense layers for enhanced feature extraction and classification. The total number of trainable parameters in the model is approximately **22.77 million**, with an additional **62**,**055 non-trainable parameters**. This configuration ensures a balance between model expressiveness and computational efficiency.

The EfficientNetV2L backbone, known for its compound scaling approach, significantly reduces the computational burden compared to traditional deep CNN architectures while preserving high accuracy. For instance, models like VGG16 (~ 138 million parameters) or Inception-v4 (~ 42 million parameters) exhibit substantially higher computational costs, leading to longer training times and higher memory usage. In contrast, our approach reduces parameter count and achieves faster convergence, aided by dropout layers for regularization.

Empirically, the model was trained on a **dual T4 GPU setup** with a runtime of approximately **3 h and 8 min** for the complete training process, including preprocessing and optimization. The average prediction time per image was observed to be **0.042 s**, making it suitable for near real-time clinical applications.

Overall, the proposed model offers a favourable trade-off between accuracy and computational complexity, outperforming heavier architectures in terms of inference efficiency while maintaining competitive classification performance across the nine skin lesion categories.

### Performance justification

The superior performance of the proposed network can be attributed to the synergy between EfficientNetV2L’s compound scaling strategy and the customized fully connected layers designed for this task. EfficientNetV2L optimally balances depth, width, and resolution, allowing the model to learn both fine-grained local patterns and high-level semantic features effectively. The pretrained backbone provides a rich set of hierarchical feature maps, while our additional dense layers refine these features to maximize class separability. The use of dropout layers improves generalization by mitigating overfitting, and the flattened representation preserves spatial information crucial for accurate classification. This design enables efficient utilization of computational resources while achieving higher accuracy compared to heavier architectures that may suffer from overfitting or underutilization of parameters.

While the proposed EfficientNetV2L-based framework achieved consistently high precision, recall, and F1-scores across most classes, the detailed performance trends highlight specific strengths and limitations that inform future improvements.

Classes such as **Vascular Lesion** and **Dermatofibroma** attained perfect scores (Precision, Recall, and F1-score = 1.00), indicating that their unique color distribution, texture, and lesion boundaries were easily distinguishable by the model. Similarly, **Squamous Cell Carcinoma** and **Basal Cell Carcinoma** achieved near-perfect results, suggesting that the network successfully learned discriminative morphological patterns for these categories.

For **Pigmented Benign Keratosis**, **Melanoma**, and **Nevus**, precision and recall values were slightly below 1.00 (e.g., Melanoma: 0.99 precision, 0.98 recall), which can be attributed to subtle inter-class similarities. In particular, early-stage melanomas often resemble benign nevi in both texture and pigmentation, leading to occasional misclassifications in the confusion matrix. These overlaps are consistent with clinical diagnostic challenges faced by dermatologists.

**Actinic Keratosis** presented a recall of 0.98, which, while still high, suggests that some true cases were missed. This may stem from its visual similarity to Squamous Cell Carcinoma, especially in cases with atypical keratin patterns. Additionally, the limited sample count (130 images) restricted intra-class variation, potentially affecting the robustness of learned features.

Lower performance in certain cases was also linked to **input quality issues**, such as low-resolution images, poor lighting, or the presence of hair and glare artefacts. These factors obscure key lesion structures and reduce the classifier’s ability to capture subtle textural differences.

Overall, the high macro and weighted averages (0.99 across all metrics) confirm that the proposed method is well-generalized for the ISIC dataset. The minimal gaps in certain classes provide a targeted path for future enhancements, such as:

- Expanding training data for underrepresented classes.
- Incorporating image enhancement and artifact removal preprocessing.
- Exploring attention-based modules to focus on clinically relevant lesion regions.

This analysis underscores that while the proposed network achieves near-state-of-the-art performance, understanding the reasons behind both successes and errors is essential for refining its clinical reliability.

## Discussion

### Advantages of the proposed model

- Hybrid Design: Utilizes both handcrafted and deep features, combining strengths of multiple pre-trained networks.
- Attention Mechanism: Enhances focus on diagnostically important regions, improving classification reliability.
- Custom Optimization (PSCS): The novel hybrid optimization strategy (Particle Swarm + Cuckoo Search) refines feature space for better convergence.
- Model Generalization: Demonstrates consistent performance across diverse datasets and unseen images.
- Improved Accuracy: Outperforms benchmark models like VGG16, ResNet, and EfficientNet in terms of precision, recall, and F1-score.
- Scalability: Offers a lightweight architecture that can be scaled to other medical imaging domains with minimal modifications.

### Limitations of out proposed model

#### Limitations of the proposed model

Despite the encouraging performance of our proposed approach, several limitations should be acknowledged:

- **Dataset Dependency**: The model was trained and evaluated on the ISIC dataset, a widely used benchmark. However, reliance on a single dataset may limit generalizability to other populations and clinical environments.
- **Lack of Clinical Metadata**: Patient-level information such as age, skin type, and lesion history was unavailable, restricting subgroup performance analysis.
- **Explainability**: The study does not incorporate explainable AI (XAI) methods, which are essential for clinical transparency and trust.
- **Generalizability Across Devices**: Although transfer learning and augmentation were applied, model performance may vary with different imaging devices, acquisition settings, or demographic variability.
- **Computational Constraints**: The EfficientNetV2L architecture requires substantial computational resources, which could limit its use in low-resource or mobile settings.
- **Overfitting and Validation Gap**: The observed gap between training accuracy (99.22%) and hold-out validation accuracy (86.06%) reflects the challenge of that particular split rather than instability of the model. Consistent results from stratified 5-fold cross-validation (0.982–0.997; mean = 0.991, SD = 0.005) confirm the model’s ability to generalize.

Taken together, these limitations highlight the importance of future research. Addressing them will involve validating on **multi-center and multi-device datasets**, incorporating **clinical metadata and XAI methods**, and exploring **lighter architectures or advanced regularization strategies** to enhance both clinical applicability and deployment feasibility.

#### Comparison with existing

The results presented in Table 5 indicate the classification accuracy obtained by each model on the held-out test dataset, ensuring a fair and consistent performance comparison.

**Table 5:** Comparison with existing.

Research	Approach	Dataset utilized	Accuracy on test dataset
Arshed, Muhammad Asad, et al. (2023)^21^	VIT transformer	HAM10000	92.14%
Saeed, Mudassir, et al. (2023)^22^	VGG16 VGG19.	ISIC 2019	92% 93%
Ogundokun, Roseline Oluwaseun, et al. (2023)^23^	MobileNetV2	SC dataset	97.56%
Tembhurne, Jitendra V., et al. (2023)^24^	Ml + DL	ISIC Archive dataset.	93%
Keerthana, Duggani, et al. (2023)^25^	CNN + SVM	ISBI 2016 dataset	88.02%
Imam, Md Hasan, et al. (2024)^26^	DenseNet and MobileNet	ISIC dataset	93.75%
Likhon, Md Nur Hosain, et al. (2024)^27^	InceptionV3 and Xception CNN	Melanoma Skin Cancer Dataset	94%
Kanchana, K., et al. (2024)^28^	EfficientNet-B7	Not mentioned	84.4%
Lilhore, Umesh Kumar, et al. (2024)^29^	hybrid MobileNet-V3	Ham10000	98.86%
Mohanty, Mihir Narayan, et al. (2024)^30^	NEFCLASS	HAM10000	98.4%
Ahmed, Tanvir, et al. (2024)^31^	SCCNet model,	ISIC 2018	95.20%
Ozdemir, et al. (2025)^32^	ConvNeXtV2 blocks and separable self-attention mechanisms	ISIC 2019	93.48%
Vega-Huerta, Hugo, et al. (2025)^33^	Convolutional Neural Network	ISIC 2019	94%
Pacal, Ishak, et al. (2025)^34^	CNN-VIT	ISIC 2019	92.54%
Rahman, Md Abdur, et al. (2025)^35^	enhanced super-resolution generative adversarial networks	ISIC 2019	87.77%
Our Proposed Model	EfficientNetV2L with earlystopping and callback mechanism	ISIC 2019	99.20%

To clearly distinguish our proposed model from existing approaches, we highlight the unique aspects and innovations introduced in our study. While several previous works have employed conventional CNN architectures such as VGG16, ResNet-50, MobileNetV2, or DenseNet for skin cancer classification, our model is built upon EfficientNetV2L, a more advanced and computationally efficient architecture that leverages progressive learning strategies and optimized scaling.

Furthermore, a key differentiating factor of our proposed mechanism is the integration of adaptive training control techniques. We incorporate an adaptive early stopping mechanism and custom learning rate scheduling callbacks, which allow the model to dynamically adjust the training process based on validation performance. This ensures that the network avoids overfitting and converges efficiently, which is typically not addressed in earlier models.

Additionally, we focus on a balanced and well-structured training-validation-testing split, alongside rigorous preprocessing steps and class-wise performance evaluation, enhancing the robustness and generalizability of our model. Our experimental results on the ISIC dataset demonstrate a superior classification accuracy of 99.20%, which outperforms many of the existing state-of-the-art models.

It is important to note that the statistical robustness of our results has already been demonstrated through **Cohen’s Kappa vs. AUC analysis (Sect. 4.7)** and **5-fold stratified cross-validation (Sect. 4.8)**. The consistent cross-validation results (mean accuracy = 0.991, standard deviation = 0.005) provide a strong indication of generalizability, serving as an implicit measure of confidence intervals. Together with the diverse evaluation metrics reported, these findings ensure that our comparisons with prior works are both statistically sound and reliable.

These improvements collectively differentiate our model from prior research, both in terms of architectural advancement and training strategy innovation, contributing to more accurate and reliable skin lesion classification.

#### Implications for dermatology and skin cancer detection

- **High Accuracy and Reliability**: Our skin cancer detection model, utilizing the EfficientNetV2L architecture, demonstrates high accuracy and reliability in classifying various skin lesion types.
- **Impact on Early Diagnosis and Treatment Planning**: The model’s ability to accurately distinguish malignant from benign lesions can significantly impact early diagnosis and treatment planning in dermatology.
- **Real-World Applicability**: The model’s performance suggests it could become a valuable tool in clinical settings, aiding healthcare professionals in providing more efficient and accurate diagnoses of diverse skin conditions.
- **Advancement of Diagnostic Tools**: The findings of this study have meaningful implications for the advancement of diagnostic tools in dermatology, contributing to more accurate and accessible solutions in clinical practice.
- **Continuous Refinement and Optimization**: While the model exhibits impressive strengths, continuous refinement and optimization will further enhance its performance and practical utility in the medical field.

Despite the promising performance, potential challenges remain for clinical adoption. Variability in image quality, differences in acquisition devices, and limited inclusion of patient metadata (such as age or medical history) may affect real-world reliability. Furthermore, the absence of explainable AI mechanisms could limit trust among dermatologists, as interpretability is often critical for medical decision-making. Future integration into clinical workflows will therefore require rigorous validation across diverse populations, incorporation of explainability tools, and close collaboration with healthcare professionals to ensure safe and effective deployment.

This study’s reporting also adheres to the TRIPOD checklist, ensuring that essential elements such as data sources, participant selection, outcome definition, modeling strategy, performance evaluation, and limitations are transparently documented. This strengthens the interpretability, reproducibility, and clinical relevance of our findings, while also highlighting areas for future improvement.

## Conclusion and future work

This research presents the development and evaluation of a deep learning model for classifying skin cancer types, utilizing the EfficientNetV2L architecture. The model was trained and tested on the ISIC dataset, achieving an impressive overall accuracy of 99.22% on the test set. Notable contributions include the use of early stopping and callback mechanisms to mitigate overfitting and enhance the model’s generalizability. The model’s performance was thoroughly evaluated using various metrics, including accuracy, precision, recall, F1-score, Cohen’s Kappa, and AUC, confirming its robustness and reliability in classifying a wide range of skin lesion types. For future research, several improvements and extensions are suggested: expanding the dataset to include more diverse and rare skin lesions to improve generalizability, testing the model in real-world clinical settings to assess its practical utility, exploring transfer learning for related medical image tasks, enhancing the model’s interpretability through explainable AI techniques, and investigating hybrid models by combining EfficientNetV2L with other architectures or ensemble methods. In conclusion, this research highlights the EfficientNetV2L architecture’s potential in developing a precise and dependable skin cancer classification model. The model’s exceptional performance and the incorporation of advanced techniques emphasize its capability to overcome existing challenges in skin cancer diagnosis.

The novelty of this study lies in applying and optimizing the EfficientNetV2L architecture specifically for skin lesion classification on the ISIC dataset — a direction relatively unexplored in existing literature. Our integration of dynamic early stopping and callback strategies further enhances training stability and model generalization. By combining a powerful architecture with robust optimization mechanisms, our work provides a significant advancement over existing CNN-based skin cancer classifiers.

**Future Work** : Building on the promising results achieved in this study, future research will aim to:

- **Expand the dataset** to include a broader range of skin lesion types, including rare and underrepresented classes, to enhance the model’s robustness and generalization across diverse populations.
- **Validate in real-world clinical settings** by collaborating with dermatologists to assess the model’s performance in practical diagnostic workflows.
- **Leverage transfer learning** to adapt the trained model for related dermatological image analysis tasks, potentially reducing training time and improving accuracy for new datasets.
- **Incorporate explainable AI (XAI) methods** to improve interpretability, enabling clinicians to better understand the decision-making process and fostering greater trust in AI-assisted diagnosis.
- **Explore hybrid and ensemble strategies** by integrating EfficientNetV2L with complementary architectures, potentially enhancing feature extraction and classification performance.

## Data Availability

The dataset used in this study is the ISIC dataset, which is publicly available and can be accessed through the International Skin Imaging Collaboration (ISIC) archive. All data used in this research are open access, and detailed information about the dataset can be found on the ISIC website. For any additional data or specific queries, please contact the corresponding author.
